# Microfluidic system for near-patient extraction and detection of miR-122 microRNA biomarker for drug-induced liver injury diagnostics

**DOI:** 10.1063/5.0085078

**Published:** 2022-04-18

**Authors:** Maïwenn Kersaudy-Kerhoas, Antonio Liga, Appan Roychoudhury, Marilena Stamouli, Rhiannon Grant, Damaso Sanchez Carrera, Holger Schulze, Witold Mielczarek, Wilna Oosthuyzen, Juan F. Quintana, Paul Dickinson, Amy H. Buck, Nicholas R. Leslie, Jurgen Haas, Till T. Bachmann, James W. Dear

**Affiliations:** 1 Institute of Biological Chemistry, Biophysics and Bioengineering, School of Engineering and Physical Sciences, Heriot-Watt University, Edinburgh, Scotland; 2 Infection Medicine, College of Medicine and Veterinary Medicine, University of Edinburgh, Edinburgh, Scotland; 3 Centre for Cardiovascular Science, Queen Mary Research Institute, College of Medicine and Veterinary Medicine, University of Edinburgh, Edinburgh, Scotland; 4 School of Biological Sciences, Ashworth Laboratories, University of Edinburgh, Edinburgh, Scotland

## Abstract

Drug-induced liver injury (DILI) results in over 100 000 hospital attendances per year in the UK alone and is a leading cause for the post-marketing withdrawal of new drugs, leading to significant financial losses. MicroRNA-122 (miR-122) has been proposed as a sensitive DILI marker although no commercial applications are available yet. Extracellular blood microRNAs (miRNAs) are promising clinical biomarkers but their measurement at point of care remains time-consuming, technically challenging, and expensive. For circulating miRNA to have an impact on healthcare, a key challenge to overcome is the development of rapid and reliable low-cost sample preparation. There is an acknowledged issue with miRNA stability in the presence of hemolysis and platelet activation, and no solution has been demonstrated for fast and robust extraction at the site of blood draw. Here, we report a novel microfluidic platform for the extraction of circulating miR-122 from blood enabled by a vertical approach and gravity-based bubble mixing. The performance of this disposable cartridge was verified by standard quantitative polymerase chain reaction analysis on extracted miR-122. The cartridge performed equivalently or better than standard bench extraction kits. The extraction cartridge was combined with electrochemical impedance spectroscopy to detect miR-122 as an initial proof-of-concept toward an application in point-of-care detection. This platform enables the standardization of sample preparation and the detection of miRNAs at the point of blood draw and in resource limited settings and could aid the introduction of miRNA-based assays into routine clinical practice.

## I. INTRODUCTION

Despite accumulating evidence suggesting that blood microRNAs (miRNAs) are promising biomarkers, their detection for this purpose has not yet been successfully introduced into clinical practice. Roadblocks to clinical adoption include differences in preparation and extraction methods used across studies and the lack of agreed sample processing guidelines and standards.

Extracellular blood miRNAs are promising biomarkers for a wide range of diseases, but their measurement remains time-consuming, technically challenging, and expensive.^1,2^ Mature miRNAs are small non-coding RNA sequences of 19–25 nucleotides, which predominantly post-transcriptionally regulate gene expression by interacting with 3′ untranslated regions (UTRs) of target mRNAs.^3^ Circulating cell-free miRNAs can be detected in serum and plasma bound to carrier proteins such as Argonaute (Ago2),^2,4,5^ high-density lipoprotein (HDL),^6^ or encapsulated in extracellular vesicles. Certain miRNAs have organ specificity or are up-regulated with disease, which creates utility as blood-based biomarkers for human diseases.

An emerging application of miRNA-based diagnostics is drug-induced liver injury (DILI). More than 600 drugs causing liver injury have been listed in the NIH LiverTox database.^7^ Paracetamol is the commonest cause in the Western world; overdose being a very common reason for attending hospital (∼100 000/year in the UK), which results in substantial annual hospital bed occupancy (∼60 000 beds) and cost (∼£50m). In the US, paracetamol overdose accounts for more than 82 000 hospital attendances and around 450 deaths every year. New biomarkers are needed to stratify patients and improve patient care. In commercial drug development, DILI is a major impediment to new compounds reaching the market and a frequent reason for market withdrawal after launch, which can result in huge financial loss for the developer. MicroRNA-122 (miR-122) has been identified as a sensitive and specific serum DILI marker for use in paracetamol and non-paracetamol DILI.^8^ At first presentation to hospital, the quantification of miR-122 accurately identifies patients who will become ill despite current optimal therapy (clinical utility). The Clinical Liver Safety Assessment Best Practices Workshop stated in 2014 that “miR-122 could be revolutionary in the assessment of liver safety in clinical trials, potentially enabling accurate assessments from small study populations with more limited durations of study drug exposure.” Reflecting this commercial potential, in 2016, the U.S. Food and Drug Administration (FDA) and European Medicines Agency (EMA) supported the use of miR-122 in drug development, underlining its potential role in drug safety assessment.

Several technical issues still need to be addressed to ensure that miRNA-based analysis faithfully reflects the true disease state. Variables that may impact analytical outcomes include (i) the type of additive or anticoagulant in the blood collection tube, (ii) sample processing times or temperatures, (iii) hemolysis of the sample, and (iv) sample storage conditions.^9,10^ The lack of stability of miRNA in plasma and serum following several freeze–thaw cycles and in different storage conditions has been reported in several studies,^11,12^ and some evidence suggests that the duration of time taken between blood collection and processing affects the overall miRNA concentration. The general recommendation is to process samples as quickly as possible; however, there is no convenient way to reliably and reproducibly process whole blood samples at the site of blood draw. Furthermore, sources of errors in laboratory procedures are often traced to the sample preparation and pre-analytical steps. To circumvent these issues and eliminate human factors, stabilization prior to transport and storage has been proposed in the form of cell stabilization tubes, but sample preparation remains a major bottleneck in the development of point-of-care devices, with very few systems integrating even basic functions such as blood plasma separation (BPS).

**FIG. 1.**
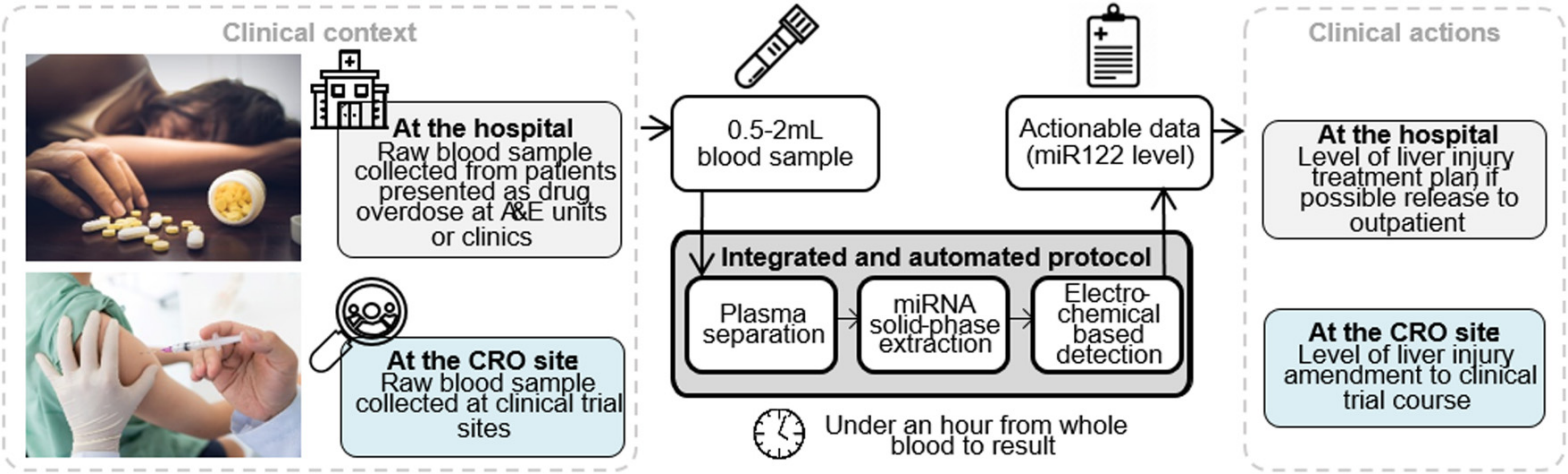
Concept of an integrated and automated protocol to reduce the time to result of microRNA-based diagnostics.

Progress has been shown in on-chip miRNA isolation from extracellular vesicles using magnetic beads and pneumatic forces in an integrated microfluidic system by Cheng *et al*.^13^ In another example, thermoelectric lysis of cells with native gel-electrophoretic elution (GEE) was demonstrated on extract synthetic miRNAs from cancer cells.^14^ The literature is rich with studies demonstrating direct detection of miRNAs.^15^ Most recently, a surface-functionalized PDMS microfluidic device reported the extraction of miRNAs from minute amounts of plasma (10 *μ*l), and in another sample, a paper device was used to detect, but not extract, miR-150-5p from just 5 *μ*l of sample.^16,17^ However, the level of analytes from capillary samples is more variable than in venous blood samples, making diagnostics less sensitive and specific.^18^ Point-of-need miRNA-122 detection would represent a rapid toxicity assay suitable in early phase pharmaceutical studies as well as in the clinic. To perform the miRNA extraction from blood draw, we developed an overall conceptual architecture with a first module for plasma extraction from blood, followed by a solid-phase extraction of plasma nucleic acids and an electrochemical based detection [Fig. 1]. This approach could be used with a number of protocols where the fast extraction of low-quantity plasma miRNA biomarkers is crucial, including the diagnosis of DILI. The article is divided into four independent modules of work, which are very briefly described here to clarify the reading.

• *Module* 1 focuses on the investigation of miRNA expression changes after blood draw, conventional centrifugation, and microfluidic blood plasma separation.
• *Module* 2 deals with the development of a microfluidic cartridge without embedded reagents (called “dry” cartridge) and has two parts:
  o the dry cartridge design and operation and
  o the dry cartridge demonstration of miRNA extraction from plasma.
• *Module* 3 describes the development of a microfluidic cartridge with embedded reagents (called “wet” cartridge) and has three parts pertaining to
  o the wet cartridge design and operation,
  o the wet cartridge demonstration of miRNA extraction from blood, and
  o the wet cartridge demonstration of miRNA detection with electrochemical detection.
• *Module* 4 focuses on the proof-of-concept of the complete workflow with clinical samples.

## II. MATERIALS AND METHODS

### A. Blood plasma separation chip fabrication

The blood plasma separation module was designed in-house alongside published microfluidic separation principles^19^ and custom manufactured by Epigem (Redcar, UK). The blood plasma separation module was composed of several layers of negative photosensitive proprietary SU8 epoxy resin forming a single layer microfluidic network with a thickness of 20 *μ*m sandwiched by a 0.5 mm PMMA substrate and a cover.

### B. miRNA extraction cartridge fabrication

The “dry” (no stored reagents) and “wet” (with stored reagents) extraction cartridges were manufactured in-house by laser cutting on an Epilog Mini18 (Epilog Laser, Clevedon, UK), layers of poly(methyl-methacrylate) (PMMA) cast sheets (Clarex, Weatherall, UK) of different thicknesses (2–0.2 mm) and bonded together, using a 2 min solvent-assisted thermal process, as previously described.^20^ The CAD files of the cartridges are available online (https://doi.org/10.6084/m9.figshare.19164860 and https://doi.org/10.6084/m9.figshare.19165301) alongside detailed Standard Operating Procedures to build them (https://doi.org/10.6084/m9.figshare.19165349 and https://doi.org/10.6084/m9.figshare.19165355). Before use, the cartridges were exposed to UV light (CX2000, Analytic Jena, Germany) for 20 min to remove DNA contamination. For the wet cartridges, the reagents were pre-loaded onto the platforms using pipets through access holes, which were then sealed using adhesive tape. A photographic step-by-step loading guide is available in Fig. S1 in the supplementary material. In this work, the wet cartridges were used straight after reagent loading. For future use, the cartridge would be pre-loaded and transported to the site of use.

### C. Sample collection

In module 1 (Sec. III A) and module 3 (Sec. III E), blood samples were collected from healthy volunteers. Ethical approval for this study was provided by the East of Scotland Research Ethics Service REC 1 committee (Reference 19/ES/0056). In the module 2 (Sec. III C) and module 3 (Sec. III F), the cartridges were tested using human plasma from the Scottish National Blood Transfusion Services (Contract 18–20). Ethical approval for this study was provided by HSC REC A (Reference 18/NI/0148). For the module 4 study with patient samples (Sec. III G), patients were recruited to the Royal Infirmary of Edinburgh (RIE), UK, for a prospective, observational, cohort study of participants aged 16 years or older. Ethical approval for this study (MAPP2) was provided by the London—South East Research Ethics Committee (Reference 18/LO/0894) (ClinicalTrials.gov identifier: NCT 03497104). Patients presenting to the RIE following acetaminophen overdose, who met the inclusion criteria, were asked to provide informed consent to participate in the study, and their demographics and blood results were recorded. The study inclusion criteria were (i) age 16 years and older, (ii) hospital attendance with acetaminophen overdose alone or as part of a mixed overdose, and (iii) patient able to give informed consent. The exclusion criteria were (i) patient detained under the Mental Health Act (Scotland), (ii) inability to provide nformed consent, (iii) unreliable history of overdose, or (iv) prisoners. Patients who presented to the RIE with a subsequent overdose were eligible to be enrolled into the study more than once. Data collected included age, gender, past medical history, date and time of overdose, amount of acetaminophen taken, other drugs taken, date and times of all treatment including blood tests, and results of all blood tests. Ethical approval details for all studies can be consulted on the website of the UK NHS Health Research Authority: https://www.hra.nhs.uk/.

To extract plasma, blood was spun down at 1600*g* for 10 min, the supernatant fluid was collected in fresh tubes without disturbing the white blood cell layer (buffy coat), and a second 10 min spin at 12 000*g* was used to remove any cell debris. Plasma was either processed or stored frozen (−80 °C) within 6 h after blood collection unless otherwise stated for experimental purposes. Once thawed, plasma was processed directly for miRNA extraction.

### D. miRNA bench extraction protocols

As a control of miRNA recovery or interference and to obtain baseline miRNA levels from plasma, miRNA was extracted and purified using the miRNeasy Serum/Plasma kit (Qiagen) in module 3.1 with an initial starting volume of 200 *μ*l of plasma and following the manufacturer instructions. miRNAs were eluted in the final volume of 14 *μ*l RNase-free water. The RNA quality and quantification were performed on a Nanodrop 1000 spectrophotometer (Thermo Scientific, Waltham, MA, USA). For bench controls in module 3.2 and onward, miRNA was extracted and purified using the QIAamp Circulating Nucleic Acid kit (Qiagen) with an initial starting volume of 400 *μ*l of plasma and following the manufacturer instructions. miRNAs were eluted in the final volume of 65 *μ*l RNase-free water.

### E. Reverse transcription

Reverse transcription (RT) reactions were performed on all obtained extractions using the miScript II RT kit (Qiagen, Hilden, Germany) in a 5 *μ*l RT reaction: 1 *μ*l 5× miScript HiSpec Buffer, 0.5 *μ*l 10× miScript Nucleic Mix, 0.5 *μ*l miScript Reverse Transcriptase Mix, and 0.5 *μ*l of 0.1 pM synthetic cel-miR-39 (Qiagen, Hilden, Germany), to control for efficiency of RT and polymerase chain reactions (PCRs), as well as to monitor the presence of potential endogenous inhibitors derived from plasma samples. These components were combined and composed the master mix. After mixing by inversion and centrifugation, the master mix was aliquoted into 0.2 ml RNase-free strip-tubes, followed by the addition of 2.5 *μ*l input RNAs.

In module 1 and module 3, RT reactions were carried out on the Biometra thermal cycler (M-Medical) using the following conditions: 37 °C for 60 min, 95 °C for 5 min, and then held at 4 °C. cDNAs were then diluted 1:10 using nuclease-free water. Diluted RT products were stored at −20 °C prior to real-time PCR. In module 2 (Sec. III B), RT reactions were carried out on the S1000™ thermal cycler (Bio-Rad) using the conditions: 37 °C for 60 min, 95 °C for 5 min, and held at 4 °C. cDNAs were then diluted 1:5 using nuclease-free water (Qiagen).

### F. Quantitative PCR

In module 1 (Sec. III A), the real-time polymerase chain reaction (qPCR) reactions were performed in duplicate using the miScript SYBR Green PCR Kit (Qiagen). Briefly, 10 *μ*l reaction volumes were prepared in a 384-well plate using 5 *μ*l SYBR Green MasterMix, 1 *μ*l miScript universal primer (Qiagen), 1 *μ*l miRNA specific primer mix, and 2 *μ*l RT product per well. The mix was aliquoted in duplicate into RNase-free strip-tubes and sealed with an optical plug. The qRT-PCR reactions were carried out using the Lightcycler 480 (Roche, Basel, Switzerland).

In module 2 (Sec. III B), diluted RT products were processed directly to real-time PCR. The qPCR reactions were performed in triplicate using the miScript SYBR Green PCR kit (Qiagen). Briefly, 12.5 *μ*l reaction volumes were prepared in a 96-well plate using 6.25 *μ*l SYBR Green MasterMix, 1.25 *μ*l of miScript universal primer, 1.25 *μ*l miR-122 specific assay primer (Qiagen), and 1 *μ*l diluted RT product per well. The mix was aliquoted in triplicate into RNase-free strip-tubes and sealed with an optical lid. The qRT-PCR reactions were carried on the StepOnePlus System (Applied Biosystems).

For all qPCR reactions, the same cycling conditions were applied: 95 °C for 15 min followed by 50 cycles of 95 °C for 15 s, 55 °C for 30 s, and 70 °C for 30 s. The quantification cycle (Cq) is defined as the fractional cycle number at which the fluorescence passes the fixed threshold. The raw data were analyzed using the automatic cycle threshold setting for assigning the baseline and the threshold for Cq determination.

For all RT and subsequent qPCR reactions, no reverse transcription enzyme controls (NEC) and no template controls (NTC) were included for all miRNA targets to determine the level of genomic-DNA contamination and tolerable threshold cutoff criteria. Detectable signals <40 Cqs were reported for the controls, Cqs >40 were ignored if the lowest unknown Cqs were 35. When results are reported in terms of Cq, the PCR was run in one batch on the sample plate. When the results are reported in terms of concentrations, the PCR may have been run on different plates, using standard curves with synthetic miRNAs. The amounts or concentrations were calculated back from the standard curves and several batches reported together when applicable.

### G. Electrochemical impedance spectroscopy

Screen-printed gold electrodes (DRP-C223BT) were purchased from DropSens (Oviedo, Spain) and used for electrochemical impedance spectroscopy (EIS) measurements. Each strip contained a gold working electrode (diameter 1.6 mm), a silver reference electrode, and gold counter electrode tracks. miR-122 specific thiol-modified peptide nucleic acid (PNA) probe sequences were ordered via Cambridge Research Biochemicals (Cleveland, UK) and obtained from Panagene (Daejeon, South Korea). All other chemicals used in electrode functionalization and electrochemical measurements were procured from Sigma-Aldrich (Gillingham, UK). The impedance measurements were recorded in a frequency range of 0.3 Hz–100 kHz with a signal amplitude of 10 mV rms at the measured open circuit potential using an Autolab PGSTAT12 potentiostat/galvanostat electrochemical system (Metrohm Autolab, Utrecht, Netherlands). Prior to impedance measurements, bench and microfluidic cartridge-extracted samples were diluted 1:5 with phosphate buffer saline (PBS; pH 7, 10 mM sodium phosphate, 20 mM NaCl, 0.2 mM potassium ferri/ferrocyanide). Similarly, patient samples after microfluidic cartridge extraction were diluted 2.5 or 5 times with PBS (pH 7, 10 mM sodium phosphate, 20 mM NaCl, 0.2 mM potassium ferri/ferrocyanide) and used for EIS measurements. Before probe functionalization, the electrodes were electrochemically cleaned by cyclic voltammetry in a 100 mM sulfuric acid solution. The probe immobilization was conducted by exposing the cleaned electrodes with 1.5 *μ*M thiol-modified PNA + 100 *μ*M 6-Mercapto-1-hexanol + 200 *μ*M 1,6-Hexanedithiol + 5 mM Tris(2-carboxyethyl)phosphine in 50% (v/v) dimethyl sulfoxide solution for 16 h followed by blocking with 1 mm 6-Mercapto-1-hexanol solution for 2 h. The EIS measurements were performed pre- and post-hybridization with a 1 h sample incubation using the PNA probe-functionalized electrodes and the increase in charge transfer resistance (Rct) was conveyed as “EIS Signal Ratio” after dividing 1 h post-hybridization Rct by the pre-hybridization Rct (baseline measurement). The Rct values for each measurement were calculated from the fitted data of the equivalent Randles’s circuit.

### H. Statistics

Statistical significance was determined by unpaired (paired when possible) parametric Student’s t-test. p-value significance threshold was set at 0.05. When reporting on statistical significance symbols “n.s.” is used to indicate non-significance (p > 0.05), while *, **, and *** denote p < 0.05, p < 0.001, and p < 0.0001, respectively, as per conventional practice.

## III. RESULTS

### A. Module 1: Blood plasma separation

**FIG. 2.**
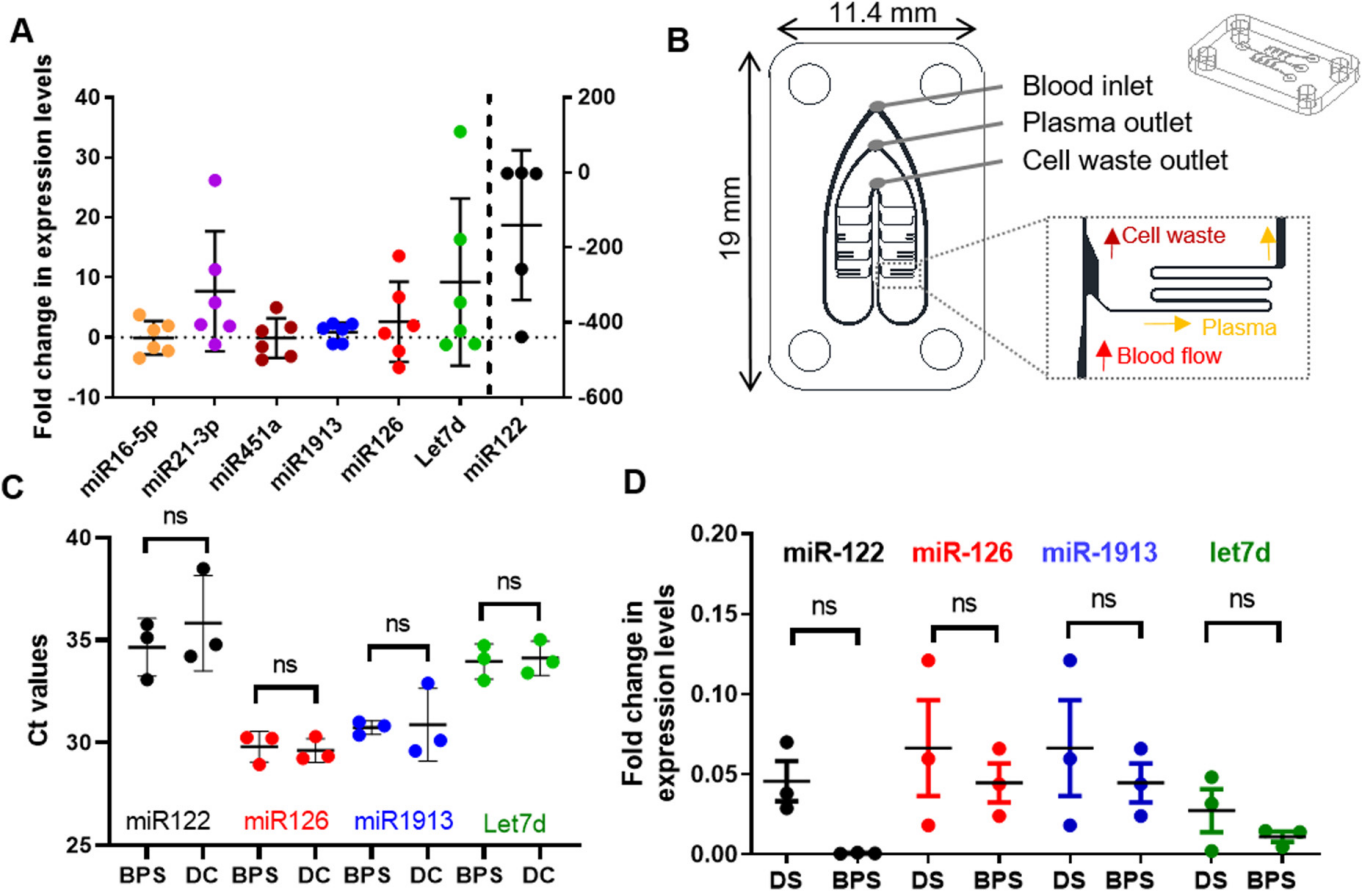
(a) Normalized fold change in a miRNA panel after same day or 48 h separation of plasma. Fold change is obtained via the ΔΔCt method using *Caenorhabditis elegans* miR-39 spike-in control. One outlier was identified at −4000-fold change for miR-122 and removed (b) Architecture of the hydrodynamic blood plasma separation (BPS) element. Details about the physical principles of similar hydrodynamic separation and its characterization have been published elsewhere.^19^ Details of a separation zone are shown in the bottom inset (dashed line). The top inset features a 3D view of the chip and detailing its overall form factor. (c) Comparisons of miRNA levels between the two plasma extraction methods: standard centrifugation protocol (DC) and blood plasma separation platform (BPS). Duplicate RT-qPCR reactions for the panel of miRNAs (miR-122, miR-126, miR-1913, and let7d) extracted by bench protocol from plasma either fractioned by standard double centrifugation or through the blood plasma separation platform. Data are expressed as the raw Ct values ± SD, N = 3. No significant differences found, paired Student’s t-test. (d) Stabilization of miRNA in separated plasma samples by centrifugation or double centrifugation. Data were normalized via the ΔΔCt method using *C. elegans* miR-39 spike-in control. N = 3. Paired Student’s t-test.

We investigated the effect of a 48 h delay in sample processing as a pre-analytical variable on a panel of representative miRNAs. Two blood samples per healthy volunteer (N = 6) were obtained. One sample was processed within 4 h after blood collection, while the second sample was stored at room temperature and processed 48 h after blood collection. In both cases, following blood plasma separation by double centrifugation (DC), plasma was frozen at −80 °C. Before amplification, plasma was thawed and miRNA isolated using Qiagen’s miRNeasy extraction. Following the extraction, an RT-qPCR was performed on the eluates. MiRNA fold change, with respect to the freshly processed samples, was calculated in absolute values as FC = 2^|*Cq*^0−^*Cq*^48|^^ (where FC is the fold change, Cq0 is the qPCR quantification cycle for sample processed at time 0, and Cq48 is the quantification cycle for the sample processed after 48 h blood incubation) and plotted in the positive axis if the marker increased and in the negative axis if marker decreased after 48 h. As shown in Fig. 2(a), after a 48-h delay, the average concentration of miR-1913, miR-126, miR-16-5p, and miR-451 did not change significantly, although intra-sample variance increased across subjects with occasional increases or decreases of the biomarker. miR-21-3p and let7d showed a general increase with processing delay. miR-122 was the one that showed the highest variability, with levels decreasing to the point that in some samples this biomarker was no longer detectable after 48 h. We show here that matched samples, with comparable initial marker levels, showed results differing up to more than two orders of magnitude after being subjected to the 48 h delay before processing. This highlights the need for rapid sample processing to obtain accurate comparisons of miRNA levels.

One of the challenges of sample preparation of circulating extracellular biomarkers such as miRNAs is the blood plasma separation step. For small volumes of blood, typically in the range of 10–100 *μ*l, a simple filtering step may be integrated on the chip; however, when biomarkers are present at low concentration, up to several milliliters of plasma may be needed to obtain a meaningful result.^21,22^ These volumes cannot generally be obtained with filtering methods due to filter saturation. We report for the first time the evaluation of a blood plasma separation module in the context of miRNA extraction. Microfluidic plasma isolation from 5 ml of blood was achieved by using a plasma separation module based on passive hydrodynamic plasma separation principles previously described.^19^ The system features a series of constrictions and bifurcations allowing the creation of cell-free zones in the blood flow on the chip and the extraction of clear plasma from side channels [Fig. 2(b)]. The channel lengths of the chip used in this study have been adapted to equalize the flow rate ratio at each bifurcation.

Figure 2(c) illustrates how a microRNA subset panel (miR-122, miR-126, miR-1913, and let7d) had comparable Ct values (qPCR performed on the same plate) after standardized double centrifugation (DC) or microfluidic blood plasma separation (BPS) indicating that the plasma quality is comparable between the two methods and that miRNAs do not bind to the microfluidic structures. Bland–Altman plots were used to visualize the agreement between the two plasma fractioning methods with miR-122, miR-126, and miR-1913 all showing a bias inferior to 1 between microfluidic blood plasmas separation and double centrifugation with only extraction of let7d diverging (bias = 1.080, SD = 1.477) (data not shown).

Finally, to investigate the stability of the plasma obtained by double centrifugation or microfluidic blood plasma extraction, miR-122 levels were measured in the plasma extracted within 2 h after plasma separation or stored for an extra 48 h prior to extraction from plasma. All four miRNAs (miR-122, miR-126, miR-1913, and let7d) were found to have only minor changes after 48 h plasma storage [Fig. 2(d)], a large improvement from the variability observed after 48 h blood storage [Fig. 2(a)]. No significant change between the double centrifugation and the microfluidic plasma separation was observed, although miR-122 showed a higher, but not significant, fold change after double centrifugation, compared to microfluidic extraction. One explanation could be that the microfluidic plasma extraction, as well as removing blood cells, could be sifting some larger extracellular vesicles containing miR-122.

### B. Module 2: Dry cartridge design and operation

**FIG. 3.**
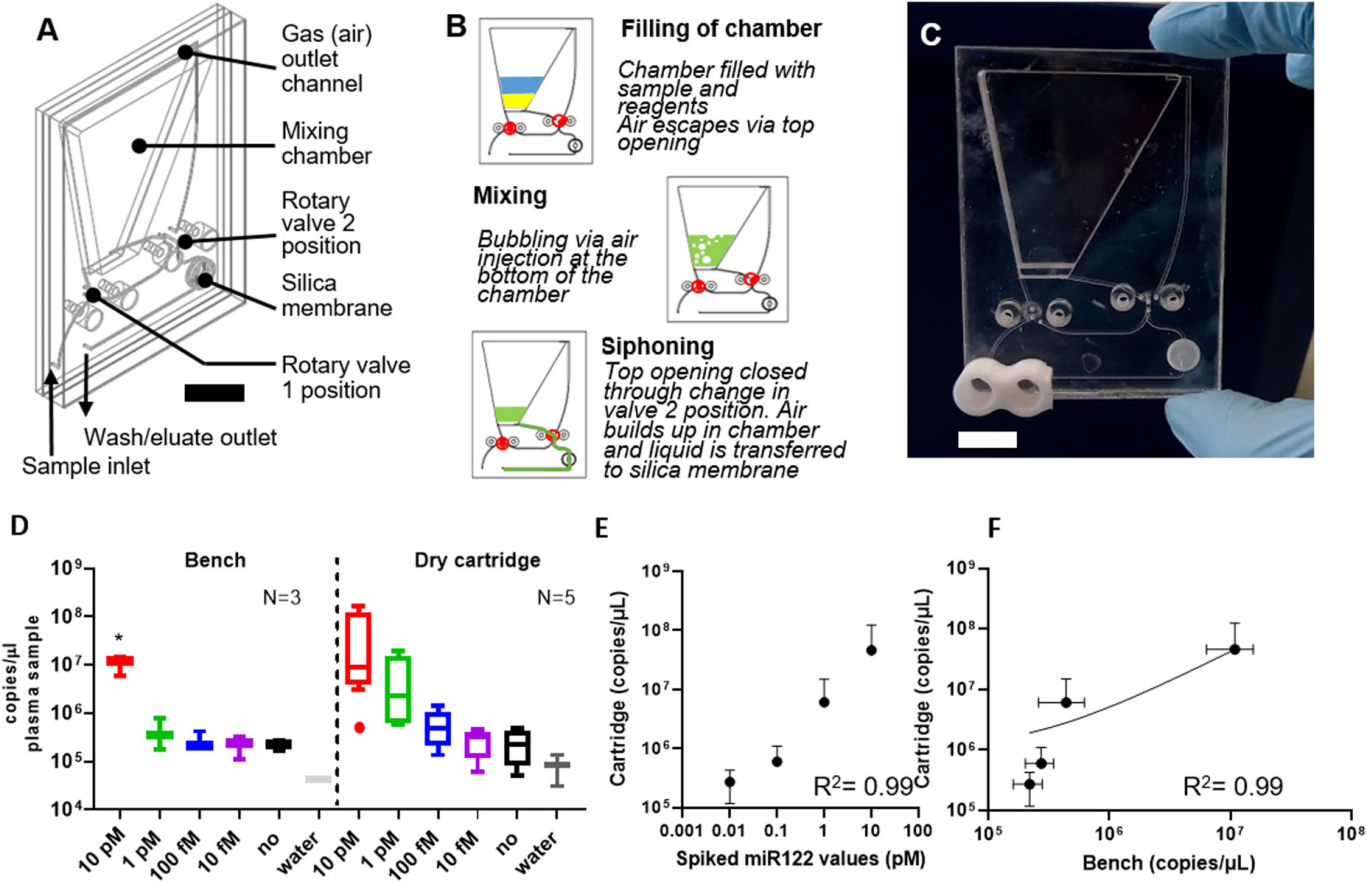
(a) Dry cartridge architecture with main elements highlighted. Scale bar is approximately 1 cm. (b) Main functional steps in the operation of the dry cartridge. (c) Photograph of a dry cartridge. Scale bar is approximately 1 cm. (d) Characterization of bench and microfluidic extraction efficiency on 10 pM, 1 pM, 100 fM, and 10 fM. Native plasma samples are indicated as “no” (no spiking) and water controls as “water.” Reported as copies/*μ*l (e) Correlation between spiked miR-122 (in pM) and recovered microfluidic in copies/*μ*l. (f) Correlation between bench and microfluidic copies/*μ*l. Due to the logarithmic axis, not all error bars can be shown.

A “dry” cartridge (=reagents not pre-loaded) designed for miRNA extraction is presented in Fig. 3(a). The dry cartridge was engineered around the principles of solid-phase extraction. The extractions were performed entirely within the disposable single-use fluidic cartridge. Initially, proteinase K, plasma sample, lysis buffer, binding buffer, and isopropanol are sequentially injected within the mixing chamber. The plasma was first lysed to release nucleic acid molecules from vesicles and other extracellular carriers. The sample was then mixed with chaotropic agents passed through a membrane that binds nucleic acid molecules. Two valves controlling the flow pattern were positioned to allow air to escape while the reagents were retained at the bottom of the chamber by gravity. Actuating the pump generated a bubble flow effectively mixing sample and reagents. After this step, the valves closed the air vent and opened the liquid outlet [Fig. 3(b)]. The air now pushed the reagent mixture through a silica membrane, where nucleic acids were adsorbed. In the last valve configuration, inlet and outlet channels were in direct communication with the membrane, bypassing the mixing chamber. After a triple washing step, a heater placed against the membrane compartment was turned on and air was flowed to dry the membrane. Finally, the elution buffer was pushed on the membrane, and after a short incubation, the air pump was activated one last time to recover the miRNA eluate in a fresh tube.

### C. Module 2: Microfluidic miRNA extraction from plasma

The dry cartridge [photographed in Fig. 3(c)] was tested using human plasma spiked with miR-122 amounts mimicking the full clinical range from high levels (10 pM) to normal levels (100 fM). RT-PCR (miScript SYBR Green PCR) performed in triplicate successfully distinguished the 10 pM–100 fM range in microfluidic and bench controls but could not distinguish a concentration of 10 fM from background miR-122 in plasma [Fig. 3(d)]. Excellent correlation between the spiked-in values and the microfluidic eluates [R^2 ^= 0.99, Fig. 3(e)] and between bench and microfluidic extraction efficiency was achieved [R^2 ^= 0.98, Fig. 3(f)]. To further optimize this technology, we developed a wet cartridge with embedded reagents and an automated protocol.

### D. Module 3: Wet cartridge design and operation

To alleviate any human errors and facilitate reagent integration, we designed a cartridge where reagents are pre-loaded “wet” cartridge and also developed a fully automated platform.

**FIG. 4.**
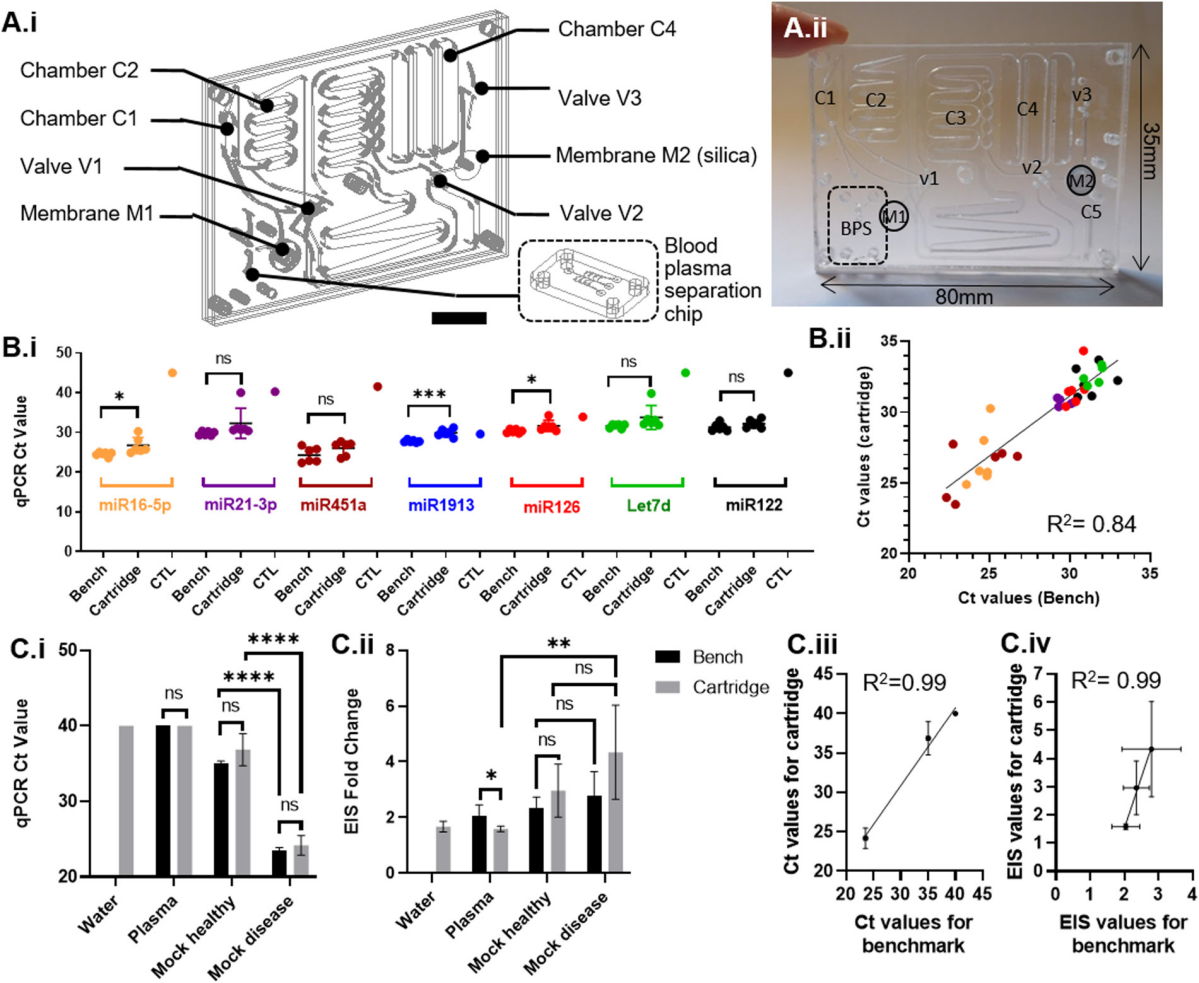
(a-i) Architecture of a wet cartridge. (a-ii) Photograph of the wet cartridge. (b-i) Comparison of Ct values for bench and wet cartridge eluates on a miRNA panel. (b-ii) Correlation of Ct values between bench eluates and microfluidic eluates for miR16-5p, miR21-3p, miR451a, let7d, and miR-122. R^2^ = 0.85. Two data points were not included in this analysis when qPCR duplicates showed Ct values differing more than 5 Cts or within 2 Cts from negative control. When including all of the miRNA panel R^2^ = 0.65. (c-i) Ct values on water, plasma, and spiked plasma. “Mock healthy samples” are donor plasma samples spiked with 10 fM miR-122 and “Mock Disease samples” are donor plasma samples spiked with 1 pM miR-122. (c-ii) Comparison of electrochemical impedance spectroscopy (EIS) results in terms of fold change for miR-122 detection with plasma-spiked microfluidic cartridge extraction. (c-iii) Correlation between bench and microfluidic Ct values. (c-iv) Correlation between bench and microfluidic EIS values.

The disposable single-use wet cartridge composed of two parts: (i) a blood plasma separation module described earlier (cf. Sec. III A) and (ii) a miRNA extraction module based on the same principle as the previously described “dry” cartridge. The nucleic acid extraction module has one inlet for the plasma emerging from the first module. Close to the inlet, there is a small 0.5 cm^2^ filter [M1 in Figs. 4(a-i) and 4(a-ii)] (Vivid GR membrane, Pall) to collect any cells, which might escape into the plasma due to the presence of a plug flow upon actuation and stabilization of the flow in the blood plasma separation module (M1). Thanks to this filter, no priming of the blood plasma module was necessary. The cartridge featured six pre-loaded reagent chambers. Chambers C1, C2, C3, C4, and C5, respectively, contain Proteinase K, lysis buffer, binding buffer, wash buffer 1, and wash buffer 2. The plasma coming out of module M1 was mixed with the necessary reagents in chambers C1, C2, and C3, and nucleic acids were adsorbed in the membrane M2 integrated within the cartridge. Air flow provided via a syringe ensured that all the volumes contained in the chambers pass through M2. Changing the valve settings in V1 and V3 allowed us to direct the airflow, first to force the wash buffers flow through the membrane, then air dry the membrane, and finally elute the adsorbed miRNA nucleic acids. Valve V2 was turned during the last step allowing to differentiate the eluate from the waste stream and to collect the eluate into a clean tube. Fluid motion within the cartridge was regulated through two syringe pumps (World Precision Instrument, Sarasota, FL, USA). The first one actuated the blood sample through the BPS module and part of the NA extraction module; the other was used to generate compressed air. Blood samples were diluted (off-chip) in PBS (Sigma) 1:1 (V/V) and pumped at 8 ml/h to actuate the blood flow into the cartridge. The actuation of the valves was controlled by an operator through a custom Labview program. V1 and V2 were turned and air flow was actuated through C5 at a flow rate of 100 ml/h by the second syringe pump after the reagents had been mixed with the samples and flowed through the silica membrane. Following this, V1 and V3 were turned to allow a fast air flow rate (550 ml/h) for 5 min in order to dry the membranes. A minute after the drying step, V1, V2, and V3 were turned so as to allow the elution buffer (10 ml/h) to pass through the membrane M2 and elute miRNAs into a fresh Eppendorf tube at the outlet.

### E. Module 3: Microfluidic miRNA extraction from blood

In order to be used in a near-patient setting, the consumable and platform must be able to process whole blood and transform it into the extracted miRNA eluate. Thus, the extraction capability of the integrated system, including BPS module and wet cartridge, was evaluated using fresh blood drawn from six healthy individuals processed within 6 h after blood collection. The microfluidic extraction was benchmarked against the gold-standard protocol (benchtop plasma separation by double centrifugation and Qiagen miRNeasy protocol GS) on a miRNA panel including miR16-5p, miR21-3p, miR-451a, miR-126, miR-1913, and let7d, as well as miR-122. Microfluidic eluates and bench eluates showed comparable levels [Fig. 4(b-i)] for miR-16-5p, miR-21-3p, miR-451a, miR-126, let7d, and miR-122 (no significant difference, or weak difference, p-values 0.0317, 0.1424, 0.1428, 0.1684, 0.1168, and 0.0470, respectively). The microfluidic and bench eluates’ miRNA measurement correlated well [R^2 ^= 0.84, Fig. 4(b-ii)]. However, miR-1913 was found to behave differently in the microfluidic eluates (p = 0.0005).

### F. Module 3: Electrochemical impedance spectroscopy (EIS) based detection of miRNA-122

The wet cartridge extraction system for miR-122 was further tested for its compatibility validated with an electrochemical impedance-based molecular detection platform. For such purposes, sequence specific binding of miR-122 was monitored using peptide nucleic acid (PNA) probe-immobilized electrodes and EIS. For this, the microfluidic cartridge extraction of miR-122 was performed on water, plasma, and contrived plasma samples mimicking healthy or diseased patients (spiked with 10 fM or 1 pM of miR-122). The cartridge extractions were run without the blood plasma cartridge and M1 filter. The same custom platform was used. Alongside cartridge extraction, bench controls were performed on all samples (Qiagen miRNeasy kit). Afterward, the cartridge and bench-extracted miR-122 were measured both with RT-qPCR and with the EIS-based assay. While the qPCR measurements enabled the identification of mock healthy vs disease samples [Fig. 4(c-i)], the EIS measurement did not achieve a high statistical difference between healthy and disease contrived samples [Fig. 4(c-ii)]. There was, however, a high statistical significance (p = 0.0034) in EIS measurements between the native plasma samples coming from healthy donors and contrived disease samples. Figures 4(c-i) and 4(c-ii) show the successful detection of miR-122 at the two different concentrations. Cartridge and bench-extracted samples exhibited good correlation on both qPCR and EIS results [Figs. 4(c-iii) and 4(c-iv)].

### G. Module 4: Clinical proof-of-concept

**FIG. 5.**
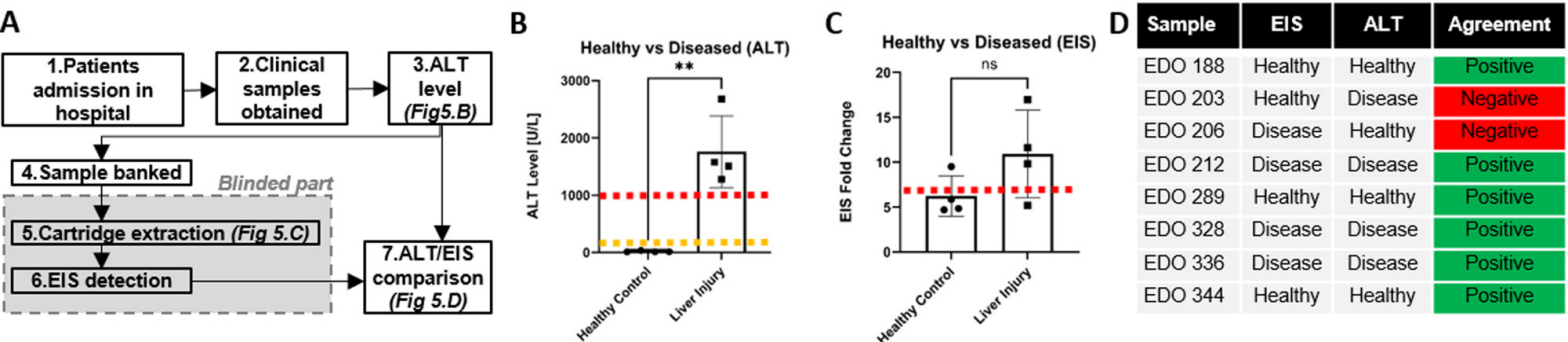
(a) Module workflow from patient admission to ALT/EIS comparison (b) ALT results on fresh samples. For ALT > 100 U/l (orange dashed line), liver injury is diagnosed, while for ALT > 1000 U/l (red dashed line), hepatotoxicity is diagnosed. (c) EIS detection of microfluidic cartridge-extracted miR-122 in liver-injured and healthy control serum samples against ALT categorization. A potential threshold value for liver injury is shown as a red dashed line. (d) Comparative table with respective ALT reference value. Six out of eight blinded EIS predictions were correct, against ALT-based diagnostic. Out of the two incorrect predictions, there is one false positive (EDO 203) and one false negative (EDO 206) corresponding to a specificity and sensitivity of 75%.

To establish a clinical proof-of-concept validation of the combined workflow, eight clinical serum samples were selected from the MAPP2 study for blinded testing using the developed microfluidic cartridge extraction platform (dry model described in Secs. III B–III E) and the EIS system (see Sec. III F). The module workflow is shown in Fig. 5(a). Briefly after admission to hospital, blood from patients enrolled in the MAPP2 trial (see Sec. II C) was sampled and alanine transaminase (ALT) activity was measured as part of routine clinical care in the local Clinical Biochemistry Laboratory. In clinical practice, ALT is commonly used to screen for hepatocellular injury.^23^ The samples were banked at −80 °C until further use. Eight samples were selected from the MAPP2 sample bank for this investigation. The frozen samples were thawed and processed using the microfluidic workflow previously described. miR-122 concentration was measured using the EIS method on the microfluidic eluates, with the aim to differentiate liver-injured samples from healthy controls. The ALT activity in the selected samples is reported in Fig. 5(b). The threshold for acute liver injury was set up at ALT > 100 U/l while hepatotoxcity was diagnosed when ALT > 1000 U/l. An EIS fold change value of 7 was set as the threshold to distinguish healthy from diseased samples [Figs. 5(c) and 5(d)]. Using this threshold, samples EDO 188, 203, 289, and 344 were determined to be healthy while EDO 206, 212, 328, and 336 were determined to be diseased. After this, the sample ALT classification was revealed by the clinical team, and the EIS result samples were further classified into healthy and diseased based on the ALT levels classification [Fig. 5(b)], which revealed satisfactory performances of the developed system except for two samples (EDO 203 and 206). In most cases, patient samples with higher miR-122 level resulted in a higher EIS fold change, whereas the healthy control samples with lower miR-122 level revealed comparatively lower EIS signal.

## IV. DISCUSSION

Studies on miRNA stability in blood have shown significant variation in miRNA levels after 48 h incubation in blood.^11^ Solutions are needed for the immediate extraction of miRNA biomarkers in applications such as the diagnosis of drug-induced liver injury. One of the challenges of sample preparation of circulating extracellular biomarkers such as miRNAs is the blood plasma separation step. For small volumes of blood, typically in the range of 10–100 *μ*l, a simple filtering (dead-end or other) step may be integrated on chip;^24–26^ however, in the situation where biomarkers are present at low concentration, several milliliters of plasma may be needed to obtain a meaningful result.^21^ These volumes cannot generally be obtained with filtering methods due to filter saturation. Thanks to an integrated hydrodynamic solution, the system presented here was demonstrated to continuously extract plasma from several milliliters of blood without clogging. We have shown that the proposed microfluidic blood plasma separation module is able to deliver plasma with the same miRNA quality (no significant fold change after 48 h storage) with double centrifugation from several milliliters of blood, without the need of a centrifuge and batch processing, allowing for seamless integration in a microfluidic workflow. A current limitation is the need for dilution, but an integrated solution may be added in the future.

Following plasma extraction, microRNAs have to be purified prior to their use in an analytical workflow such as quantitative PCR, electrochemical impedance spectroscopy, and Next Generation Sequencing (NGS). Two microfluidic approaches were proposed in this work. In module 2 (Secs. III B and III C), a semi-automated solution where each step was controlled by the operator was demonstrated. In this approach, reagents were introduced one by one into the workflow. The dry cartridge was successfully demonstrated to detect varying concentrations of spiked miR-122.

A fully automated workflow from start (crude sample) to finish (microRNA eluate) was demonstrated on whole blood or plasma in module 3 and module 4 (Secs. III D–III F). The system was capable of alleviating any human errors compared to the manual conventional bench protocols. Currently, the microfluidic protocol takes 45 min when starting from 5 ml of 1:1 diluted whole blood, compared to the 2 h of trained staff manual work required for the full bench protocol (including the double centrifugation step). While a large number of microfluidic-based solutions for cellular and extracellular (cell-free) nucleic acid extraction have been proposed,^27,28^ there are only a few demonstrations of miRNA extraction on-chip, although some direct quantification has been proposed by others.^29–31^

Next, the microfluidic sample preparation workflow was combined with EIS measurements to confirm the compatibility of the extraction with an analytical assay and assess the level of miR-122 after microfluidic cartridge extractions. EIS is efficient in studying bio-recognition events by investigating interfacial properties at the modified surfaces. This method uses small amplitude perturbation from steady state, which provides a non-destructive approach.^32^ EIS has the advantage of not requiring labels, as compared to other amplification-free miRNA detection methods such as fluorescence-based,^33^ amperometry,^34^ or voltammetry.^35^ This simplifies sensor development and operation. Furthermore, EIS is advantageous in terms of simplicity and ease in miniaturization as compared to other optical methods, e.g., surface plasmon resonance sensor.^36^ Moreover, EIS readout allows fast and sensitive detection with minimal steps using simple handheld instrumentations,^37,38^ which is most suitable for scaling up for wider use and thus for the development of an effective point-of-care test (POCT) device. Water, native human plasma, and plasma samples spiked with two different concentrations of miR-122 mimicking healthy and diseased states (10 fM and 1 pM) were used in the developed cartridge. After extraction, the miR-122 level was directly detected using the specific probe-functionalized electrodes and EIS without any amplification. The plasma samples spiked with 10 fM and 1 pM of miR-122 provided higher EIS signal as compared to the blank plasma owing to the hybridization of extracted targets. Furthermore, the 1 pM spiking samples showed higher EIS responses as compared to the 10 fM spiking samples (p > 0.05 seen as not significant). The EIS measurements performed in quadruplicate for 1 pM spiking samples successfully discriminated from the blank plasma measurements (p < 0.0001), but the results could not distinguish 10 fM spiking samples from the background plasma with a statistically significant difference (p > 0.05). For the bench-extracted plasma samples, the EIS responses were marginally higher for 10 fM spiking samples from the background plasma and further higher for 1 pM spiking samples (p > 0.05 seen as not significant). The EIS results were compared with standard qPCR analysis, which successfully distinguished both 10 fM and 1 pM spiking samples from the background plasma (p < 0.0001). A significant difference between 10 fM and 1 pM spiking samples after both cartridge and bench extractions (p < 0.0001) was observed. However, both qPCR and EIS analysis confirmed the successful extraction of synthetic miR-122 using the developed microfluidic cartridge. We further investigated the correlation between EIS and qPCR results, which provided a good correlation (cartridge samples, R^2^ = 0.89, and bench samples, R^2^ = 0.98, data not shown) after microfluidic cartridge and bench extractions.

Finally, to evaluate the clinical application of the developed microfluidic cartridge, we studied eight clinical serum samples using blind examination. With the microfluidic cartridge system, the recruited clinical serum samples were analyzed and the extracted miR-122 was directly detected using EIS platform. The extracted samples from the liver injury showed a higher EIS signal due to having higher miR-122 content, whereas the healthy control revealed a lower signal. We anticipate that the diseased samples contained higher miR-122 level due to liver injury. Later, the EIS signal of individual samples was verified at their respective ALT level, where six out of eight samples displayed equivalent EIS responses according to the ALT level. However, we grouped the EIS signals from healthy and liver-injured samples to observe the difference between two sample types. After grouping, the liver-injured sample population displayed higher signal as compared to the healthy population. Though, due to changing signals from two samples, the difference between two different populations was lower (p > 0.05). Nevertheless, miR-122 extraction using the developed microfluidic cartridge followed by detection could discriminate against patients who developed liver injury from the patients without injury.

## V. CONCLUSIONS

The short stranded miR-122 molecule is a novel marker of drug-induced liver injury, which could be used at point-of-care to triage patients with life-threatening conditions. miR-122 has shown higher specificity to DILI compared to the conventional ALT and aspartate transaminase (AST) markers. There is a need to demonstrate miR-122 measurements at the point-of-care. In this work, we have demonstrated the combination of a microfluidic sample preparation module and an electrochemical impedance spectroscopy approach enabling the measurement of miR-122 at the point-of-care on a short timescale, typically around an hour. This workflow was demonstrated on a subset of clinical sample enabling the identification of disease vs healthy samples with promising results (sensitivity and specificity 75%), paving the way for a near-patient drug-induced liver injury diagnostic test.

## SUPPLEMENTARY MATERIAL

A photographic step-by-step guide of the buffer loading procedure in the wet cartridge is available in Fig. S1 in the supplementary material.

## Data Availability

CAD files of the dry and wet cartridges are available at https://doi.org/10.6084/m9.figshare.19164860 and https://doi.org/10.6084/m9.figshare.19165301, respectively. CAD files are accompanied with detailed fabrication SOPs: https://doi.org/10.6084/m9.figshare.19165349 and https://doi.org/10.6084/m9.figshare.19165355.
